# Integrative single-cell atlas unveils heterogeneity and prognostic value of cancer-associated fibroblasts in gastric cancer

**DOI:** 10.3389/fonc.2025.1559489

**Published:** 2026-01-09

**Authors:** Ziyu Jiang, Xiaomei Zhuang, Junkui Guo, Minyi Zhu, Chunhong Hong, Chunhui Sun, Kaiming Wu, Haofan Yin, Cuncan Deng, Ping Jiang

**Affiliations:** 1 Guangzhou First People’s Hospital, School of Medicine, South China University of Technology, Guangzhou, Guangdong, China; 2 Department of Endocrinology, Guangzhou First People’s Hospital, South China University of Technology, Guangzhou, Guangdong, China; 3 The Seventh Affiliated Hospital, Sun Yat-sen University, Shenzhen, Guangdong, China; 4 Digestive Diseases Center, The Seventh Affiliated Hospital, Sun Yat-sen University, Shenzhen, Guangdong, China; 5 Department of Medical Laboratory, Shenzhen People’s Hospital (The Second Clinical Medical College, Jinan University; The First Affiliated Hospital, Southern University of Science and Technology), Shenzhen, Guangdong, China; 6 Department of Clinical Laboratory, Guangzhou First People’s Hospital, School of Medicine, South China University of Technology, Guangzhou, Guangdong, China

**Keywords:** gastric cancer, cancer-associated fibroblasts, single cell, biomarker, prognosis

## Abstract

Gastric cancer (GC) exhibits molecular heterogeneity and diverse immune cell infiltration patterns closely associated with patient prognosis. However, a comprehensive understanding of the variations in immune cell phenotypes among different patient subgroups still needs to be improved. In this study, we performed a detailed analysis of the tumor microenvironment in GC by integrating 200,466 single cells from 72 patients across six datasets. We classified patients into immune-deserted, B, T, and myeloid cell subtypes. Using genomic and clinical data from TCGA samples, we identified cellular components associated with tumor histology and genotypes. GC patients were stratified into immune-deserted, B cell, T cell, and myeloid cell subtypes, and we described the pathway and transcription factor activity characteristics of different microenvironment subtypes. Integration of bulk RNA-seq data reveals that fibroblasts and endothelial cells were associated with adverse patient outcomes whereas NK and T cells were notably correlated with improved prognosis. Subsequently, we focused on characterizing cancer-associated fibroblasts (CAFs) and discovered that they acquire new functional properties within the tissue microenvironment, providing evidence of CAF plasticity. We constructed a novel four-gene CAF signature including SPARC, EFEMP1, RGS5 and SERPINE1 which may enhance patient stratification and prognostic prediction of GC patients. qPCR analysis revealed that the significant expressions of SPARC, EFEMP1, RGS5 and SERPINE1 were significantly upregulated in gastric cancer tissues compared to the normal tissues. Our study provides insights into the composition of the tumor microenvironment and construction of a four-gene CAF signature associated with clinical prognosis, offering new perspectives for the clinical management of gastric cancer.

## 1 Introduction

Gastric cancer (GC) is a severe digestive tract malignancy with high incidence and mortality rates globally. Approximately 1.03 million new cases of GC and around 800,000 deaths are reported worldwide annually (1, 2). The incidence and mortality rates are exceptionally high in the Asian region, notably in countries such as China, South Korea, and Japan (1). GC exhibits significant heterogeneity at various levels, including molecular and clinical phenotypic levels (3–5). This heterogeneity is observed among different patients and within different regions of the same patient’s tumor (6). Such heterogeneity poses challenges for treating GC and complicates our understanding of its etiology and progression mechanisms. In-depth exploration of GC heterogeneity can provide valuable insights into the underlying mechanisms of GC development, paving the way for developing novel therapeutic approaches.

In GC research, the role of the tumor microenvironment has garnered significant attention. Studies have demonstrated that the tumor microenvironment plays a crucial role in the tumorigenesis, progression, and metastasis of GC and substantially impacts treatment responses (7–9). Stromal cells constitute a vital component of the tumor microenvironment, capable of secreting various growth factors and cytokines such as VEGF and TGFβ, thereby promoting the proliferation and migration of GC cells (10, 11). Immune cells also form an integral part of the tumor microenvironment and can impact GC cells’ malignant phenotype and drug sensitivity through interactions. For instance, immune cells like macrophages (12) and T lymphocytes (13) can secrete cytokines and growth factors that exert inhibitory or stimulatory effects on the growth and proliferation of GC cells. Research on the tumor microenvironment in GC has emerged as a pivotal area of investigation, which holds the potential to unearth novel therapeutic targets, offering fresh strategies and approaches to enhance the survival rates and quality of life for GC patients.

Fibroblasts constitute a major cellular component in the tumor microenvironment and play a pivotal role in the initiation and progression of GC (14, 15). Fibroblasts, through their interactions with tumor cells, secrete cytokines and modulate the composition and structure of the extracellular matrix, thereby influencing GC cells’ proliferation, migration, invasion, and drug sensitivity. In GC, fibroblasts can be categorized into two main types: cancer-associated fibroblasts (CAFs) and normal fibroblasts (NFs). CAFs represent the predominant fibroblast subtype in tumor tissues and exhibit the closest interactions with tumor cells. Compared to NFs, CAFs promote the proliferation and migration of GC cells by secreting various cytokines and growth factors, such as IL-6 (16), IL-8 (17), and TGF-β (18). These factors activate signaling pathways in GC cells, modulating their proliferation and differentiation. Additionally, CAFs can enhance the invasion and metastasis of GC cells by regulating the composition and structure of the extracellular matrix (19). Investigating the mechanisms underlying fibroblast function holds promise for identifying novel therapeutic targets, distinguishing different types and stages of GC, and providing a basis for personalized treatment strategies.

This study systematically analyzed the tumor microenvironment heterogeneity in GC by integrating publicly available single-cell sequencing data. GC patients were stratified into immune-deserted, B cell, T cell, and myeloid cell subtypes, and we described the pathway and transcription factor activity characteristics of different microenvironment subtypes. Furthermore, by integrating publicly available transcriptomic data and clinical information, we established associations between gene expression and phenotypes, revealing that fibroblast content is most relevant to adverse prognosis in GC patients. We further analyzed the features and differentiation trajectories of fibroblasts in GC, constructed a prognostic model based on CAF-specific gene signatures, and validated its reliability across multiple datasets.

## 2 Materials and methods

### 2.1 Datasets collection

The Log2-normalized gastric cancer FPKM expression matrix of The Cancer Genome Atlas (TCGA) and the corresponding clinical and phenotype information were downloaded from the UCSC Xena official website (https://xenabrowser.net/datapages/). The gastric cancer sample expression chips GSE13861, GSE26899, GSE26901, and GSE28541 were downloaded from the Gene Expression Omnibus (GEO) database (https://www.ncbi.nlm.nih.gov/geo/query/acc.cgi). The gastric cancer single-cell datasets GSE134520 (7 samples), GSE150290 (47 samples), GSE163558 (9 samples), GSE167297 (5 samples), and GSE183904 (36 samples) were downloaded from the GEO database. The gastric cancer single-cell dataset HRA000051 (12 samples) was downloaded from the Genome Sequence Archive (GSA) database (https://ngdc.cncb.ac.cn/gsa-human/browse/HRA000051).

### 2.2 Preprocessing of single-cell datasets

All analyses were performed in R (v4.4.2) using Seurat (v5.1.0) as the primary framework. We performed quality control for each sample individually. Quality control removes low-quality data, including damaged or dead cells, empty droplets where no cells were captured, and doublets where more than one cell was captured. Generally, low-quality cells or empty droplets contain few genes, while doublets are likely to have more genes detected. Also, low-quality or dead cells will likely have more mitochondrial gene expression. Filtering criteria 1: Cells with a minimum number of expressed genes >300. Filtering criteria 2: Mitochondrial/ribosomal gene ratio <20%. Gene counts were normalized with Seurat’s LogNormalize (scale factor = 10,000), and highly variable genes (HVGs) were selected with FindVariableFeatures (vst, 3,000 HVGs). Since single-cell sequencing expects each barcode label to correspond to a single actual cell, but in practice, there may be cases where two or more cells share a barcode, which we call doublets, we used DoubletFinder (20) to identify and remove potential doublets from the analysis. To minimize the confounding effects of cell cycle-related variation on clustering, we applied Seurat’s standard cell cycle regression approach. Specifically, cell cycle scores for S phase and G2/M phase were calculated based on canonical marker gene sets (including MKI67, TOP2A, and others defined in Seurat), and these scores were regressed out during data scaling. This procedure ensures that proliferating cells are not removed but that cell cycle-associated transcriptional programs do not dominate the clustering structure. Finally, we retained 200,466 single cells from 72 patients across six datasets. For cross-study integration, we used Seurat’s RPCA-based workflow: HVGs were passed to SelectIntegrationFeatures (3,000 genes), PCA was computed per object, FindIntegrationAnchors was run with reduction=“rpca” and dims=1:50 (using a reference dataset), and IntegrateData generated a joint “integrated” assay. To further mitigate residual study-level effects, we applied Harmony on the PCA embeddings with Dataset as the covariate and used the Harmony embeddings for downstream steps. Dimensionality reduction was performed with UMAP (and t-SNE for visualization) on Harmony dimensions 1-15. Neighborhood graphs were constructed with FindNeighbors on the same dimensions, and communities were identified with FindClusters (resolution=0.6).

### 2.3 Odds ratios calculation

To assess the tissue distribution preferences of meta-clusters, we adopted the odds ratio (OR)–based approach described by Zheng et al. (21). For each combination of meta-cluster i and tissue j, we constructed a 2 × 2 contingency table that included (1): the number of cells from meta-cluster i in tissue j, (2) the number of cells from meta-cluster i in all other tissues, (3) the number of cells from non-i meta-clusters in tissue j, and (4) the number of cells from non-i meta-clusters in other tissues. The OR derived from this table reflects the relative enrichment or depletion of meta-cluster i in tissue j. An OR > 1 indicates that meta-cluster i is more prevalent in tissue j compared to other tissues, whereas an OR < 1 indicates reduced prevalence. Given the very large number of cells analyzed, statistical testing (e.g., Fisher’s exact test) often yielded extremely significant p-values even for subtle differences. Therefore, we focused on the magnitude of the OR itself as the primary indicator of tissue preference, independent of p-values.

### 2.4 Tumor microenvironment-based subtype

Based on the merged single-cell expression matrix from six datasets, we annotated cell subpopulations and calculated the proportion of each cell subpopulation in each sample. Next, we clustered all samples and their corresponding cell subpopulation proportion matrix using the hclust function. We classified the samples into immune-deserted, B cell, T cell, and myeloid cell subtypes based on the enrichment of each cell type within each cluster.

### 2.5 Integration of single-cell TME classes with TCGA-STAD clinical and molecular annotations

Because single-cell RNA-seq data inherently lack comprehensive clinical annotations, while the TCGA cohort contains rich clinical information but is not directly matched to our single-cell samples, we devised a strategy to establish a link between the two datasets. To minimize sample-to-sample variation in cell numbers, we first randomly sampled an equal number of cells from each single-cell specimen and aggregated them to generate pseudo-bulk expression profiles. Within each TME class, we identified marker genes through differential expression analysis and defined the top up-regulated genes as class-specific signatures. TCGA bulk RNA-seq data were normalized, and z-scored across samples, and for each TCGA case we computed the mean expression of each class signature. Each case was assigned to the TME class with the highest score, thereby establishing a correspondence between single-cell-derived classes and the TCGA cohort. The resulting class assignments were then integrated with clinical annotations, including Lauren classification, pathological stage, and molecular subtype. Associations between inferred TME classes and clinical categories were assessed by contingency analysis and Fisher’s exact test, and visualized using alluvial (Sankey) diagrams to highlight enrichment patterns and the relationships between TME composition and clinical features.

### 2.6 Pathway activity estimation

Based on the merged single-cell expression matrix from six datasets, we extracted the expression matrix for subsequent analysis from the Seurat object. We downloaded the cancer hallmark pathways and Kyoto Encyclopedia of Genes and Genomes (KEGG) pathways and their corresponding gene information from the Gene Set Enrichment Analysis (GSEA) database (22). Using the R package GSVA (23), we performed single-sample GSEA (ssGSEA) analysis on each sample based on the expression matrix. We considered the enrichment score of each pathway in each sample as the pathway activity level.

### 2.7 Transcription factor activity score

Based on the merged single-cell expression matrix from six datasets, we extracted the expression matrix for subsequent analysis from the Seurat object. We downloaded the regulatory relationships between transcription factors and their substrates from the GSEA database and their corresponding gene information (GTRD subset of TFT). Using the R package GSVA, we performed single-sample GSEA (ssGSEA) analysis on each sample based on the expression matrix. We considered the enrichment score of each transcription factor in each sample as the transcription factor activity level.

### 2.8 Scissor analysis

The Scissor algorithm (24) represents an innovative approach for single-cell data analysis, leveraging an extensive array of phenotypic information to identify highly phenotypically related cell subpopulations within single-cell sequencing datasets. Notably, Scissor identifies phenotype-related cells that exhibit distinctive molecular profiles characterized by essential marker genes and relevant biological processes associated with a given phenotype. Besides, the Scissor algorithm obviates the need for unsupervised clustering in single-cell data analysis, eliminating inherent subjectivity in determining cluster numbers and resolution. Furthermore, Scissor offers a flexible framework for seamlessly integrating diverse external phenotypic data into the analysis pipeline, facilitating hypothesis-free identification of clinically and biologically pertinent cell subpopulations. In this study, we used mutation, survival, and gene expression data of gastric cancer from the TCGA database to predict the cell subpopulations most associated with specific mutations, overall survival, and progression-free survival. All parameters were used with default values.

### 2.9 CAF signature-based risk score

Using the R function FindAllMarkers, we calculated differentially expressed genes (DEGs) between CAFs and other cells. We selected the top 40 genes with the highest log2 (FoldChange) for risk model construction. Using the R package “glmnet v4.1-2”, LASSO-Cox regression analysis was performed on the expression of the 40 genes in TCGA STAD samples to determine the candidate genes. Finally, after 1000 times of modification and cross-validation, a 4-gene signature was obtained. A linear combination of characteristic gene expression was used to calculate the score for each patient. The minimum criterion determined the regression coefficient. Risk score = k1 * x1 + k2 * x2 +… + ki * xi (i = n), where i represents each selected gene, k is the regression coefficient, and x is the expression level. This paper defines the final model as risk score = 0.012 * SPARC + 0.031 * EFEMP1 + 0.044 * RGS5 + 0.166 * SERPINE1. Based on the survival optimal cutoff of TCGA data, we further classified the samples into high-risk and low-risk groups.

### 2.10 Clinical specimen collection and ethics approval

Gastric cancer and normal tissue samples were obtained from the Seventh Hospital of Sun Yat-sen University. The sample collection protocol received approval from the Sun Yat-sen University Health Science Institutional Review Board (Approval No. KY-2022-051-02). All specimens were stored at -80 °C for subsequent analysis. The clinicopathologic information of clinical specimens was showed in **Supplementary Table S1**.

### 2.11 Real-Time PCR Analysis of Hub RNAs Identified by LASSO

Several hub RNAs were identified through LASSO analysis. The expression levels of these hub RNAs were quantified using quantitative PCR (qPCR). Total RNA was extracted from gastric tissues using the AG RNAex Pro RNA reagent (Accurate Biology, CAT# AG21102) according to the manufacturer’s protocol. Complementary DNA (cDNA) was synthesized with Evo MMLV reverse transcription master mix (Accurate Biology, CAT# AG11706). qPCR was performed using the SYBR Green Pro Tag HS premixed qPCR kit (Accurate Biology, CAT# AG11701). The relative expression levels of the hub RNAs were calculated using the 2^–ΔΔCt^ method, with mRNA expression normalized to β-actin. Primer sequences for all RNAs used in qPCR are listed in **Supplementary Table S2**.

### 2.12 Bioinformatics analysis

The evaluation of epithelial cell copy number variation is implemented using the infervcnv package. The mutation landscape of TCGA gastric cancer samples is implemented using the R package maftools (25). The pseudo-time series analysis is implemented using the R package monocle3 (26). The survival analysis and KM curve plotting are implemented using the R package survival (27). The time-dependent subject work characteristic curve is implemented using the R package timeROC. The nomogram and calibration curves are calculated and drawn using the R package rms.

## 3 Results

### 3.1 Generation of a core gastric cancer single-cell atlas

**Figure 1:**
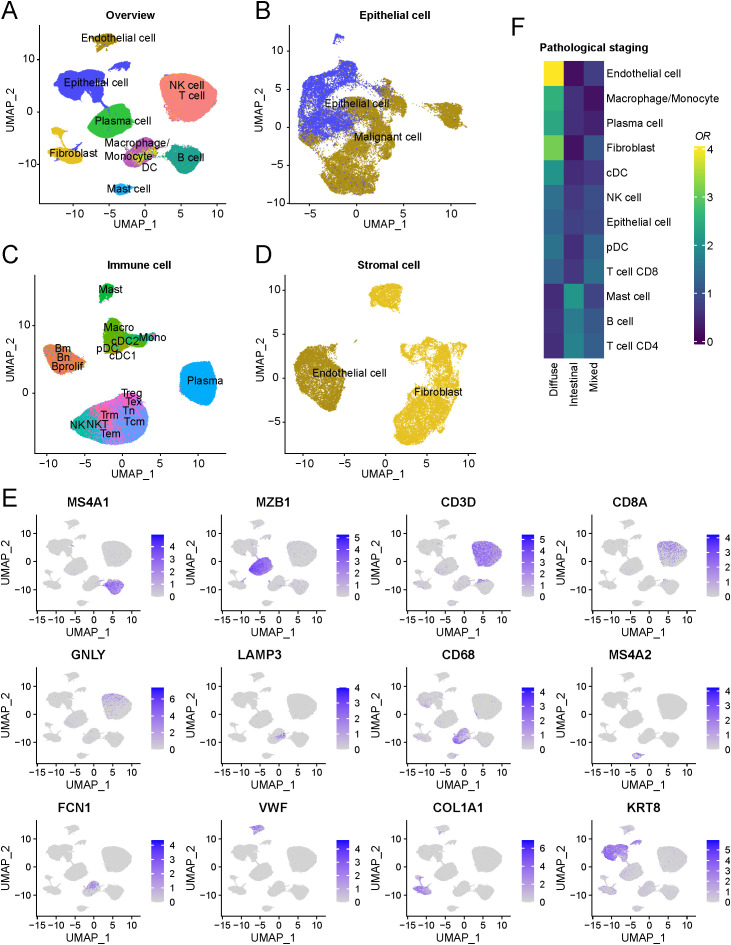
Schematic outline of the overall concept used in this study. **(A)** Overview of the core gastric cancer atlas depicted as uniform manifold approximation and projection (UMAP) plots. **(B)** Overview of the epithelial components depicted as UMAP plots. **(C)** Overview of the immune components depicted as UMAP plots. **(D)** Overview of the stromal components depicted as UMAP plots. **(E)** UMAP of cell-type marker genes used for cell-type annotation. **(F)** Cell-type composition among different clinical subtypes.

In this study, we curated publicly available single-cell sequencing data for gastric cancer. Following quality control and doublet removal procedures, we retained 200,466 single cells from 72 patients across six datasets to establish the core gastric cancer single-cell atlas. After mitigating batch effects using the harmony algorithm, no prominent batch effects were observed between different datasets, and distinct cell types could be effectively delineated (**Supplementary Figure S1A**). Through annotation with specific marker genes, we classified these cells into three major groups: epithelial cells, immune cells, and stromal cells (**Figures 1A–D**). These encompass epithelial cells, endothelial cells, cancer-associated fibroblasts, B cells, plasma cells, mast cells, macrophages/monocytes, dendritic cells, natural killer cells, and T cells. As depicted in **Figure 1E**, the markers for epithelial cells were KRT8, for endothelial cells were VWF, for cancer-associated fibroblasts were COL1A1, for B cells, were MS4A1, for plasma cells were MZB1, for mast cells were MS4A2, for macrophages/monocytes were CD68, for dendritic cells were LAMP3, for T cells were CD3D, and for natural killer cells were GNLY. We excluded cells with high expression of cell proliferation-related genes such as MKI67 and TOP2A.

Overall, T cells constituted the highest proportion of all cells, followed by epithelial and B cells (**Supplementary Figure S1B**). However, substantial variations in the proportions of immune cells and epithelial cells were observed across different dataset samples (**Supplementary Figure S1C**, **Supplementary Table S3**). By comparing samples with available tumor stage information, we found that diffuse gastric cancer exhibited notably higher proportions of endothelial cells, macrophages, and cancer-associated fibroblasts, along with lower proportions of CD4+ and CD8+ T cells (**Figure 1F**). This suggests that the enrichment of these cell types within the tumor microenvironment of diffuse gastric cancer possibly contributes to an immunosuppressive microenvironment, leading to a poorer prognosis than intestinal-type gastric cancer (28). Additionally, we compared the cellular composition differences between metastatic and primary lesions, revealing a significant reduction in dendritic cell content in metastatic lesions (**Supplementary Figure S1D**). The distinct microenvironment of metastatic lesions compared to primary lesions might arise from adverse conditions such as hypoxia (29), nutrient scarcity (30), and acidic environments (31), potentially inhibiting the growth and function of dendritic cells.

Further stratifying epithelial cells based on tissue origin and copy number variation (CNV) results, we classified them into normal and malignant epithelial cells. As shown in **Supplementary Figure S1E**, malignant epithelial cells exhibited substantially elevated CNV levels relative to normal epithelial cells. Upon comparing the expression of genes associated with normal gastric secretion and digestive functions, such as PGC, CLDN18, MUC5AC, VSIG1, TFF2, ANXA10, and CTSE, we observed markedly higher expression of these marker genes in normal epithelial cells.

### 3.2 Single-cell composition of the TME reveals distinct gastric cancer immune phenotypes

**Figure 2:**
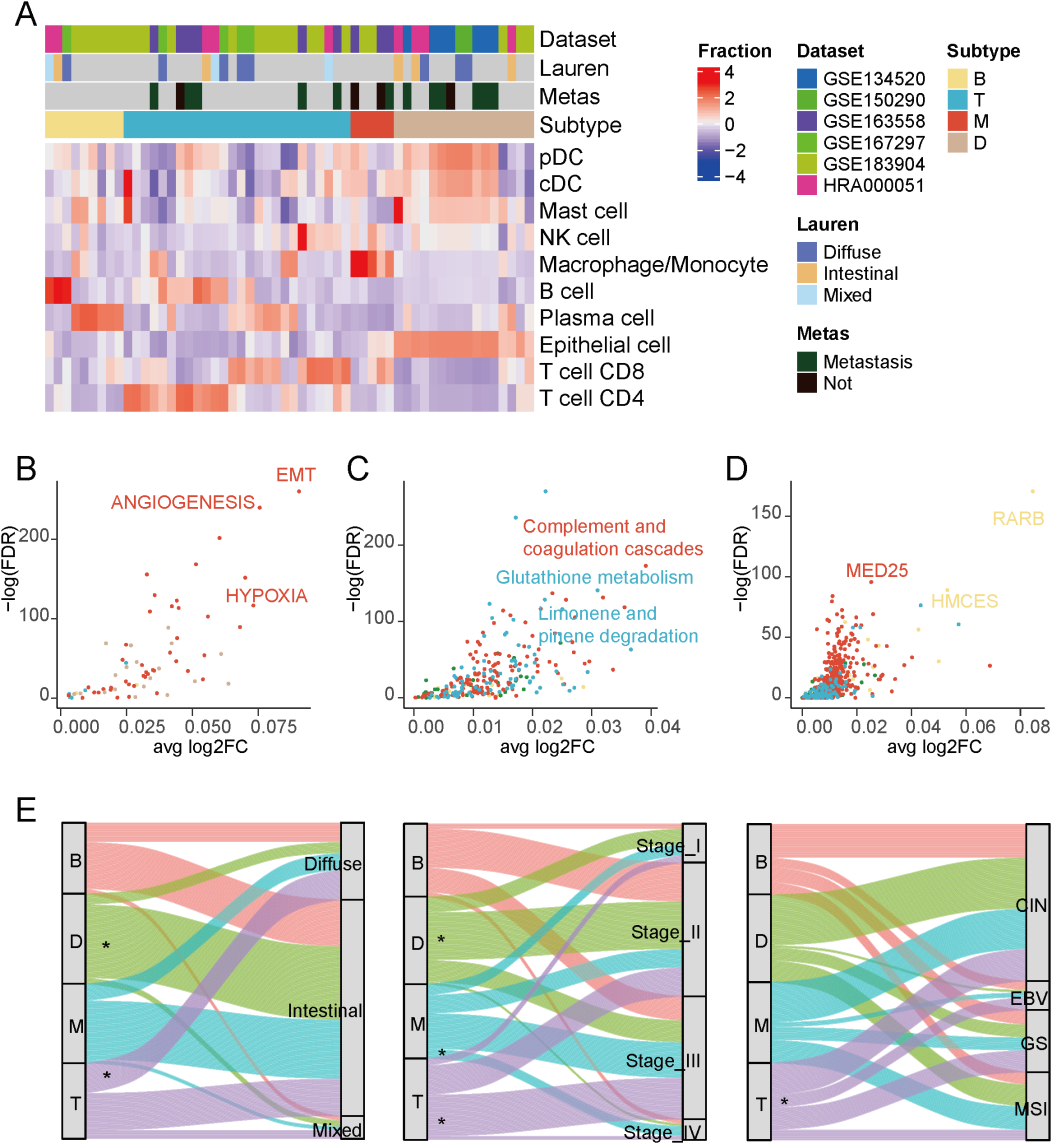
Tumor immune phenotypes in gastric cancer. **(A)** Patient characteristics and stratification of the tumor immune phenotypes. Tumor type (histopathological) refers to the histological subtypes provided by the original datasets based on pathological assessment. **(B)** Differential activation of cancer hallmark pathways between the four tumor immune phenotypes in cancer cells. **(C)** Differential activation of KEGG pathways in cancer cells between the four tumor immune phenotypes. **(D)** Differential activation of transcription factors in cancer cells between the four tumor immune phenotypes.

Next, we stratified patients into distinct groups based on each sample’s differential composition of immune cells. As shown in **Figure 2A**, we categorized patients into four types: immune-deserted, T cell-infiltrated, B cell-infiltrated, and macrophage-infiltrated, based on the differences in the enriched cell types within each subtype. The immune-deserted type (D-type) was predominantly composed of epithelial cells, while other immune cells maintained relatively lower levels. The macrophage-infiltrated (M-type) exhibited a significant increase in macrophages/monocytes. The T cell-infiltrated (T-type) displayed the highest levels of T cell infiltration, including CD4+ T cells and CD8+ T cells. The B cell-infiltrated (B-type) had a relatively high proportion of B and plasma cells (**Supplementary Figure S2**).

To discern the tumor cell gene expression characteristics in each subtype, we first compared the pathways differentially enriched in tumor cells among the different subtypes. As shown in **Figure 2B**, M-type tumor cells displayed marked epithelial-mesenchymal transition (EMT) activity, hypoxia levels, and angiogenesis activity (**Supplementary Table S4**). Studies have indicated that macrophages can secrete various growth factors and cytokines, such as vascular endothelial growth factor (VEGF), to promote neovascularization (32). They can also produce signaling molecules, such as transforming growth factor-beta (TGF-β), inducing tumor cells to undergo EMT (33), thereby increasing tumor invasion and metastasis capability. Additionally, they can generate a substantial amount of nitric oxide and other molecules, potentially leading to local hypoxia (34). Contrasting cellular metabolic pathways (**Figure 2C**, **Supplementary Table S5**), Glutathione metabolism and Limonene and pinene degradation were significantly upregulated in the T cell-infiltrated type, and some studies have suggested that the regulation of glutathione metabolism may influence the chemotaxis and recruitment of immune cells, including T cells (35).

Finally, we compared each subtype’s differentially enriched transcription factors (**Figure 2D**, **Supplementary Table S6**). We observed a significant enrichment of RARB in the B cell-enriched subgroup. RARB is involved in the differentiation processes of various cell types. Retinoic acid regulates cell differentiation status in many cell types by binding to RARB (36). RARB also plays a crucial role in the immune system, influencing T and B cells’ activation, differentiation, and function, which is essential for maintaining immune system balance and responding to infections (37). In the macrophage-enriched subgroup, we also noted the enrichment of the transcription factor MED25.

We next examined the distribution of the newly defined TME subclusters (B, T, M, and D) across clinicopathological categories in the TCGA-STAD cohort (**Figure 2E**). When stratified by Lauren classification, the D subtype was preferentially enriched in intestinal-type tumors, whereas the T subtype was more common in diffuse-type tumors, consistent with prior observations that diffuse gastric cancer is characterized by stronger T-cell infiltration. In the analysis of pathological stage, D tumors were most frequent in Stage II disease, T tumors were enriched in Stage III, and M tumors, which are macrophage-dominant, were overrepresented in Stage IV cases, suggesting a potential link between macrophage-driven microenvironments and advanced disease progression. Finally, when mapped onto the TCGA molecular subtypes, the T subtype was markedly enriched in EBV-related tumors, in line with the established immunogenic and T cell-inflamed phenotype of EBV-positive gastric cancers. Together, these results support the biological relevance of our immune-based TME subclusters and indicate that they capture distinct facets of tumor biology that align with known pathological and molecular features of gastric cancer.

### 3.3 Integration of bulk RNA-seq data reveals genotype-immune phenotype associations

In the following analysis, we leveraged the recently developed bulk transcriptome and single-cell phenotypic association tool, SCISSOR, to establish connections between cell types, genotypes, and survival status within the context of single-cell sequencing data. We aimed to uncover differences in immune cell components across various phenotypes.

**Figure 3:**
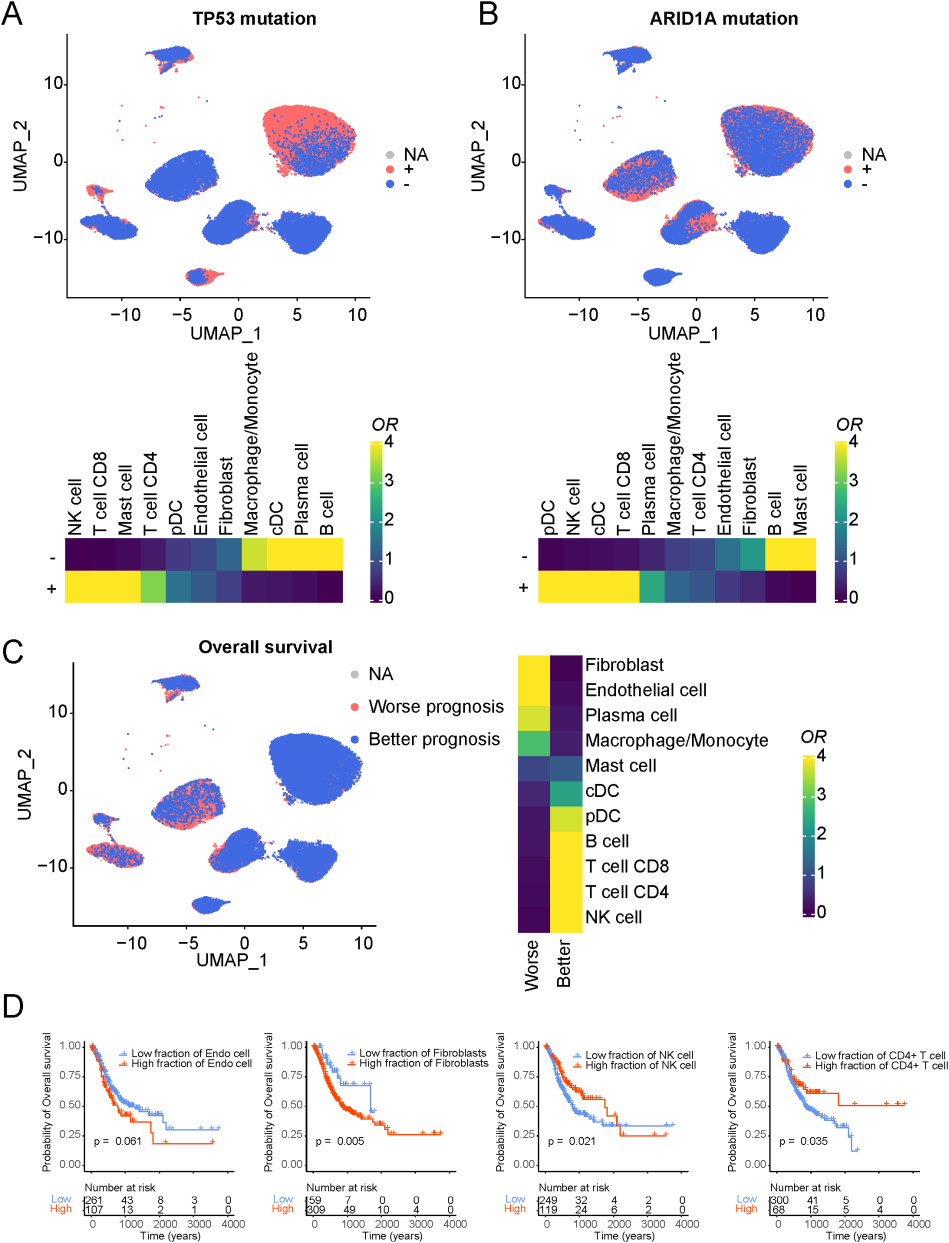
Association of cellular composition and distinct genotypes and survival in the TCGA data. **(A)** Association of cellular composition with TP53 mutation in patients with STAD. **(B)** Association of cellular composition with ARID1A mutation in patients with STAD. **(C)** Association of cellular composition with overall survival. **(D)** Kaplan-Meyer plot of patients with high and low Endothelial/Fibroblasts/NK/T cell fractions of TCGA patients with gastric cancer as determined by deconvolution with xCell. P-value has been determined using CoxPH regression using tumor stage and age as covariates.

We initially focused on the gene mutation information and their corresponding cell components. As depicted in **Supplementary Figure S3A**, we summarized some high-frequency mutations observed in gastric cancer based on TCGA mutation data. Notable mutations such as TP53, ARID1A, TTN, and the methyltransferase KMT2D were identified. Given the frequent occurrence of TP53 and ARID1A mutations in gastric cancer, which has been well-documented in the literature, we investigated their relationships with immune cell composition (**Figures 3A, B**). B cells and plasma cells exhibited a significant negative correlation with TP53 mutations, whereas NK cells and T cells showed a positive correlation. Some studies suggest that TP53 mutations inhibit CTL function by inducing Tregs amplification and IL-34-mediated macrophage M2 polarization in the tumor microenvironment (38).

Interestingly, similar observations were made in the context of ARID1A mutations, suggesting that TP53 and ARID1A mutations may contribute to an immunosuppressive microenvironment by suppressing T cell activity, thereby promoting malignant tumor progression. We also noted a significant decrease in mast cells in samples with ARID1A mutations (**Supplementary Figure S3B**). Mast cells can influence tumor cell growth and invasion by secreting various growth factors and cytokines and may assist tumors in evading immune surveillance by suppressing immune responses (39). Similar trends were observed in the context of TTN and KMT2D mutations (**Supplementary Figures S3C, D)**.

Further linking immune cell components with overall survival status (**Figure 3C**), we identified cell populations significantly associated with adverse patient outcomes, including fibroblasts and endothelial cells. Conversely, NK and T cells were significantly associated with better patient prognosis. We extended our analysis to estimate different cell components for each sample using xCell based on TCGA gastric cancer data and analyzed their relationships with patient overall survival (**Figure 3D**). Notably, the survival analysis based on bulk data and the phenotype associations based on single-cell data exhibited consistent results. Fibroblasts exhibited the strongest association with adverse patient outcomes, emphasizing the critical role of cancer-associated fibroblasts in promoting tumor initiation and progression. Furthermore, phenotype associations based on progression-free survival analysis demonstrated similar trends (**Supplementary Figure S4**).

### 3.4 Plasticity and canonical functional properties of CAFs

**Figure 4:**
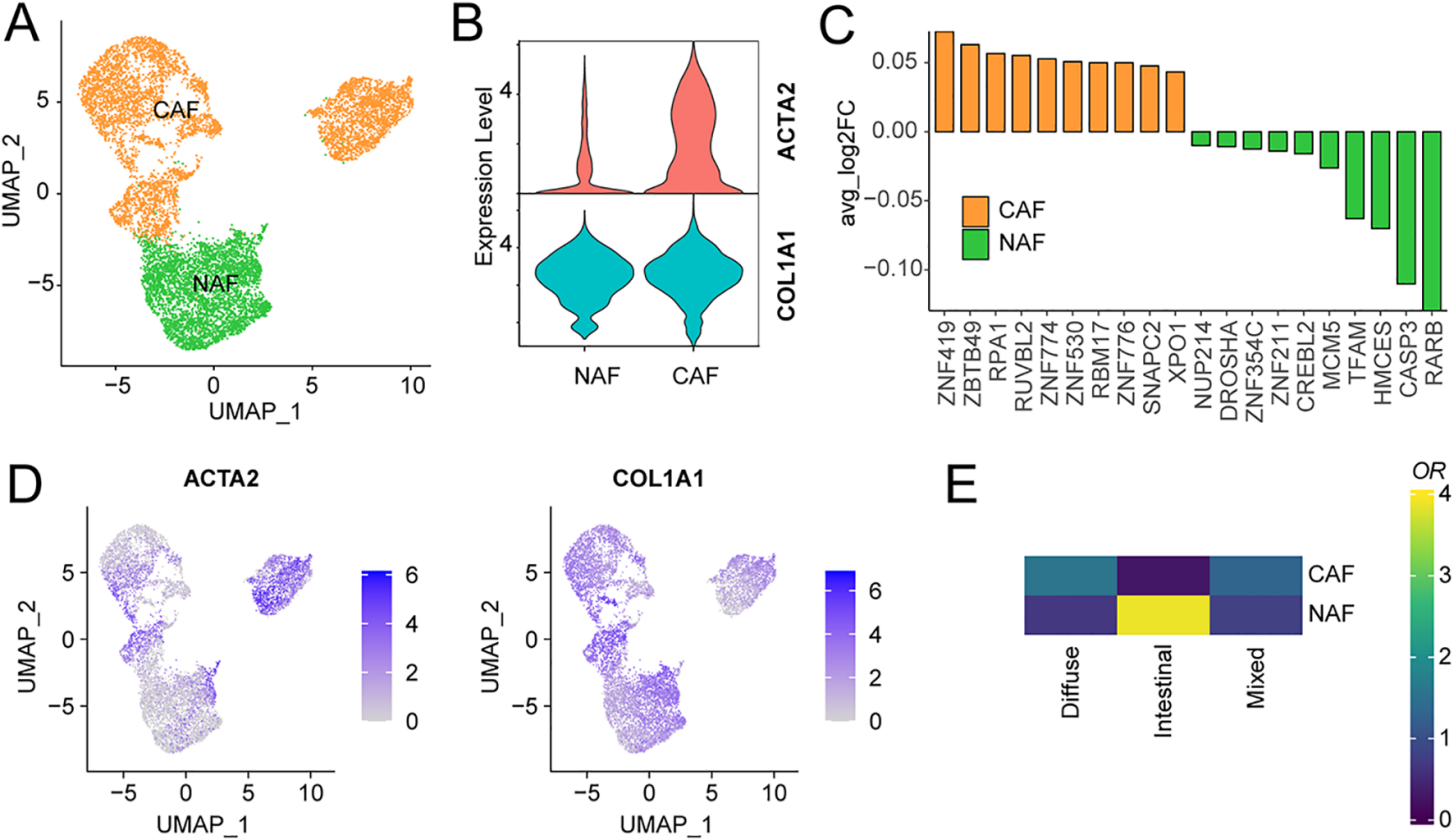
Characterization of cancer-associated fibroblasts using scRNA-seq. **(A)** UMAP of CAFs from the extended atlas was classified into cancer-associated fibroblasts (CAFs) and normal-associated fibroblasts (NAFs). **(B)** Expression levels of COL1A1 and ACTA2 between CAFs and NAFs. **(C)** Transcription factor analysis of CAFs versus NAFs using ssGSEA. **(D)** UMAP of cell type marker genes used for cell-type annotation. **(E)** Fibroblasts cell-type composition among different clinical subtypes.

Given the strong correlation between fibroblast components and overall survival in gastric cancer, we performed a comprehensive subdivision of all fibroblasts based on distinct marker expressions, classifying them into cancer-associated fibroblasts (CAFs) and normal-associated fibroblasts (NAFs) (**Figure 4A**, **Supplementary Table S7**). COL1A1 serves as a classical marker for normal fibroblasts, while ACTA2 is a common CAF marker (40), typically employed to identify activated fibroblasts actively shaping the tumor microenvironment. We observed widespread expression of COL1A1 in all fibroblasts, while ACTA2 exhibited specific and elevated expression in CAFs (**Figures 4B**, **D**). Interestingly, CAFs were notably more abundant in diffuse and mixed-type gastric cancers, whereas intestinal-type gastric cancer exhibited significantly higher levels of normal fibroblasts (**Figure 4E**). Since intestinal-type gastric cancer is associated with the best prognosis, we speculate that CAFs may play a distinctive role in influencing the prognosis of gastric cancer patients. Transcription factor activity analysis indicated a significant upregulation of ZNF419 in CAFs (**Figure 4C**). Previous studies have identified ZNF419 as a marker for immune microenvironment alterations and adverse prognosis in cancer and as a promising candidate therapeutic target (41).

**Figure 5:**
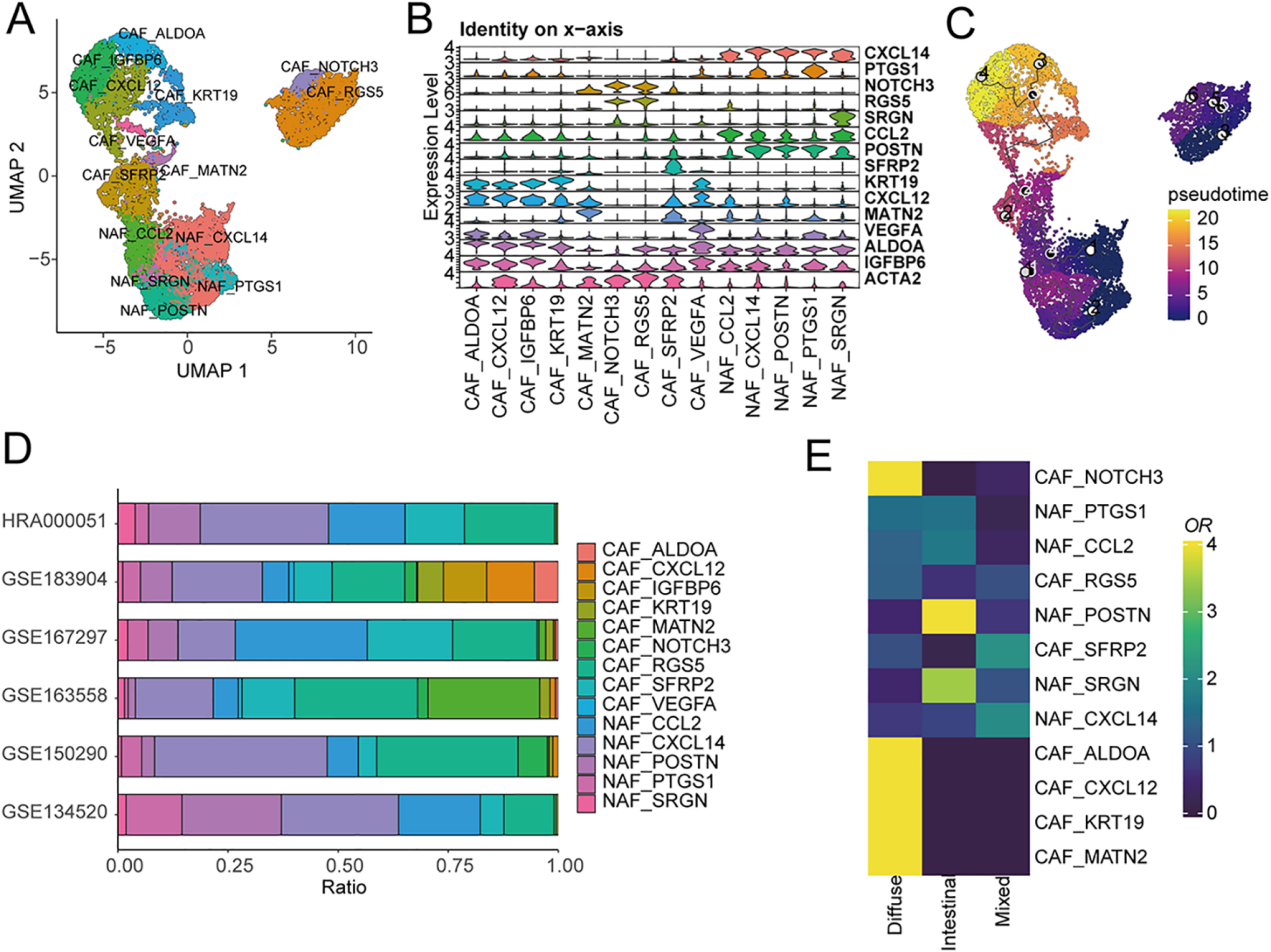
Tissue-resident fibroblasts subtypes in gastric cancer. **(A)** UMAP of all fibroblasts colored by CAFs and NAFs subclusters. **(B)** Expression levels of marker genes between CAFs and NAFs subclusters. **(C)** UMAP of all fibroblasts from the core gastric cancer atlas with monocle vectors projected on top. **(D)** Fibroblasts cell type fractions in the core atlas. **(E)** Fibroblasts subclusters composition among different clinical subtypes.

Subsequently, we further characterized fibroblasts into subtypes based on their marker expressions (**Figure 5A**). CAFs were divided into nine subtypes, while NAFs were classified into five subtypes (**Figure 5B**). Significant differences in various fibroblast subtypes were observed across different datasets (**Figure 5D**) and differentially expressed genes between different fibroblasts subclusters were showed in **Supplementary Table S8**. In diffuse gastric cancer, subtypes with high expression of ALDOA, CXCL12, KRT19, and MATN2 were significantly enriched (**Figure 5E**). Research has indicated that ALDOA may be upregulated in certain cancers, particularly within CAFs in the tumor’s surrounding tissues. ALDOA participates in the glycolytic pathway, a crucial energy-producing pathway for cells (42). In the tumor microenvironment, CAFs can influence the metabolism of cancer cells and provide them with essential metabolic substrates, thereby promoting tumor growth. The expression of CXCL12 in CAFs can affect tumor growth and metastasis. CXCL12 can recruit immune cells and other cell types, promoting their localization within tumor tissues (43). This process can influence the tumor microenvironment, subsequently impacting tumor development and treatment response. Pseudo-time analysis suggested that CAFs with high ALDOA and CXCL12 expression represent terminal subtypes differentiated from normal fibroblasts (**Figure 5C**), indicating that during the malignant progression of cancer, normal fibroblasts transform into activated cancer-associated fibroblasts.

### 3.5 CAFs gene signature is associated with a worse prognosis

**Figure 6:**
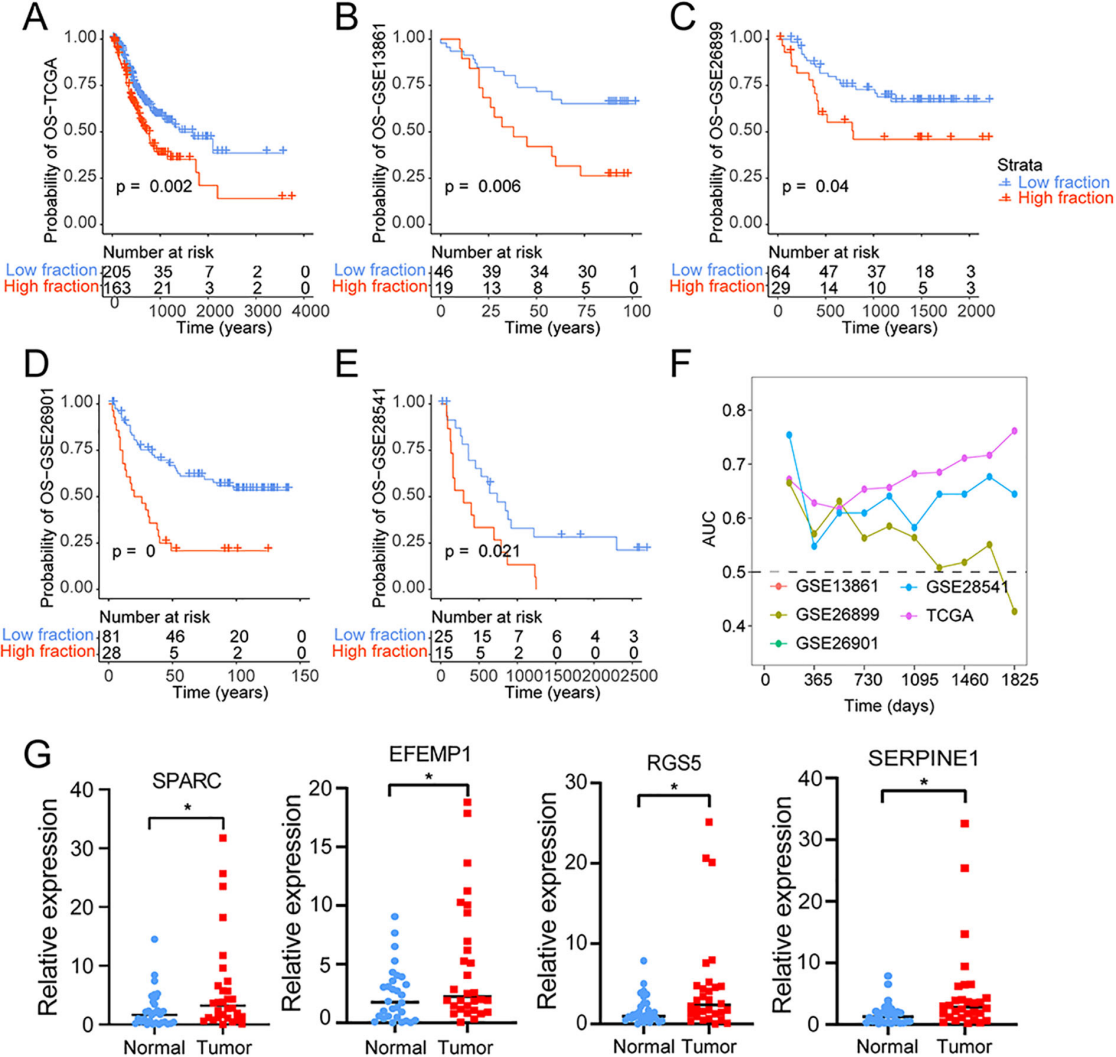
Identification of CAF-based signature and construction of risk model. **(A–E)** Kaplan-Meyer plot of patients with high and low-risk scores of **(A)** TCGA, **(B)** GSE13861, **(C)** GSE26899, **(D)** GSE26901, **(E)** GSE28541, patients with gastric cancer as determined by risk model. **(F)** Time-dependent AUC value in TCGA, GSE13861, GSE26899, GSE26901and GSE28541. **(G)** The expression of SPARC, EFEMP1, RGS5 and SERPINE1 were significantly upregulated in gastric cancer compared to the normal tissue. * means *p* less than 0.05.

We obtained single-cell gene expression profiles and subsequently identified the significantly upregulated genes within cancer-associated fibroblasts (CAFs) compared to other cell types. We selected the top 40 differentially expressed genes for subsequent analyses (**Supplementary Table S9**). To identify optimal prognostic gene biomarkers among these 40 CAF-specific genes, we applied a LASSO-Cox regression model to the gene expression profiles and clinical data from TCGA STAD samples. Subsequently, we established a four-gene signature model, with the risk score calculated as follows: risk score = 0.012 * SPARC + 0.031 * EFEMP1 + 0.044 * RGS5 + 0.166 * SERPINE1. Detailed model parameters are illustrated in **Supplementary Figures S5A**, **B**. The predicted risk score exhibited significant distinctions across various survival statuses (**Supplementary Figures S5C**, **D**). Kaplan-Meier survival analysis and Cox regression demonstrated that patients with higher risk scores had significantly poorer prognoses (**Figure 6A**). **Figures 6B–E** depict the robust performance of the risk score across different datasets, highlighting its consistency in predicting survival probabilities.

Additionally, time-dependent ROC curve analysis underscored the substantial potential of the risk score in forecasting patient survival statuses (**Figure 6F**). Furthermore, based on the risk score, we developed a nomogram to predict 1-year, 3-year, and 5-year overall survival probabilities. As depicted in **Supplementary Figure S6A**, the nomogram analysis displayed minimal deviation between the risk score and actual OS probabilities at these time points (**Supplementary Figures S6B–D**). The key genes included in the LASSO-Cox regression model (including SPARC, EFEMP1, RGS5 and SERPINE1) were significantly upregulated in gastric cancer compared to the normal tissue (**Figure 6G**).

## 4 Discussion

In this study, we conducted a comprehensive analysis of gastric cancer at the single-cell level, providing valuable insights into the intricate interplay between immune phenotypes, genotype-phenotype associations, and the pivotal role of CAFs in gastric cancer progression and prognosis.

Our establishment of a core gastric cancer single-cell atlas revealed a diverse landscape of cell types within the TME. By identifying specific marker genes, we classified these cells into three major groups: epithelial cells, immune cells, and stromal cells. The abundance of immune cells, particularly T cells, B cells, macrophages/monocytes, and cancer-associated fibroblasts, varied significantly across different samples. These variations underscore the dynamic nature of the TME and its potential impact on clinical outcomes. Our findings emphasize the crucial role of specific cell types within the TME, particularly in the context of gastric cancer subtypes. Notably, the enrichment of endothelial cells, macrophages, and cancer-associated fibroblasts in diffuse gastric cancer and their lower proportions in CD4+ and CD8+ T cells may contribute to an immunosuppressive milieu and, consequently, poorer prognosis compared to intestinal-type gastric cancer (44, 45). These observations underscore the importance of understanding the differential composition of immune cells within distinct subtypes of gastric cancer, as this knowledge could pave the way for subtype-specific therapeutic strategies.

We also delved into the intricate relationship between gene mutations, immune cell composition, and patient outcomes. Notably, TP53 and ARID1A mutations, frequently observed in gastric cancer (46, 47), were associated with distinct immune profiles. TP53 mutations exhibited a positive correlation with B cells and plasma cells but a negative correlation with NK cells and T cells. This suggests that TP53-mutated tumor cells may possess mechanisms to evade immune surveillance, thereby promoting tumor progression (48). ARID1A mutations displayed similar associations, potentially contributing to an immunosuppressive TME by suppressing T cell activity (49). Key pathways associated with immune phenotypes were identified. For instance, M-type tumor cells exhibited increased epithelial-mesenchymal transition (EMT) activity, angiogenesis, and hypoxia. These findings align with the role of macrophages in promoting tumor invasion and metastasis through the secretion of growth factors like VEGF and signaling molecules such as TGF-β (32, 33).

Furthermore, the upregulation of Glutathione metabolism and Limonene and pinene degradation pathways in T cell-infiltrated tumors underscores the potential influence of metabolic pathways on immune cell chemotaxis and recruitment. Transcription factor enrichment analysis revealed that RARB was significantly enriched in B cell-infiltrated tumors. RARB’s involvement in regulating immune system function and cell differentiation is noteworthy (50), suggesting its potential as a target for therapeutic interventions. The enrichment of MED25 in the macrophage-infiltrated subgroup highlights its role in modulating gene expression in these cells. MED25 is a key subunit of the Mediator complex, which is responsible for linking transcription factors to RNA polymerase II (Pol II) and precisely regulating the transcription of target genes through the recruitment of chromatin modifiers (e.g., histone acetyltransferase HAC1) and the promotion of chromatin loop formation (51). MED25 may regulate macrophage gene expression in infection or inflammation by enhancing the recruitment of transcription factors or stabilizing pre-transcriptional initiation complexes. MED25 may be involved in M2 polarization by regulating transcription factors such as STAT6 or IRF4 (52).

CAFs emerged as a central player in gastric cancer progression (53, 54). Our study classified fibroblasts into CAFs and NAFs based on distinct marker expressions. CAFs, marked by ACTA2 expression (40), were notably more abundant in diffuse and mixed-type gastric cancers. This observation raises intriguing questions about the distinctive roles played by CAFs in influencing patient prognosis (15), particularly in different gastric cancer subtypes (45). Among the essential genes identified in CAFs, ALDOA and CXCL12 stood out due to their potential implications in promoting tumor growth and metastasis. ALDOA’s involvement in glycolytic pathways within the TME suggests its role in fueling cancer cell metabolism (55). In contrast, CXCL12’s ability to recruit immune cells and other cell types may shape the TME, impacting tumor development and treatment response.

Limitations of this study include the reliance on publicly available single-cell sequencing datasets, which may introduce variability in data quality and experimental protocols. Gastric cancer’s inherent heterogeneity might not be fully captured through single-cell analysis alone, potentially limiting the study’s ability to represent the spatial and temporal complexities within the tumor microenvironment. While key genes and pathways associated with immune phenotypes and prognosis were identified, functional validation is required to confirm their roles in gastric cancer progression. Moreover, the study’s clinical data could be enriched with more comprehensive patient information. In terms of further plans, it is essential to conduct experimental validation of the identified genes, such as TP53, ARID1A, ALDOA, and CXCL12, and translate these findings into clinical trials to evaluate potential therapies. Integrating spatial transcriptomics (56), combining scRNA-seq with proteomics, epigenomics, conducting longitudinal studies, and incorporating multi-omics data will provide a more comprehensive understanding of the disease. Additionally, validating the nomogram in larger patient cohorts and exploring therapeutic target development will contribute to advancements in gastric cancer research and clinical care.

## 5 Conclusions

A comprehensive understanding of the variations in immune cell phenotypes among different patient subgroups still needs to be improved. This study aims to reveal distinct gastric cancer immune phenotypes and construct a novel prognostic panel.We classified patients into immune-deserted, B, T, and myeloid cell subtypes. We constructed a novel four-gene CAF signature including SPARC, EFEMP1, RGS5 and SERPINE1 which may enhance patient stratification and prognostic prediction of GC patients.Our study provides insights into the composition of the tumor microenvironment and construction of a four-gene CAF signature associated with clinical prognosis, providing new perspectives for the clinical management of gastric cancer.

## Data Availability

All bulk transcriptomic, single-cell transcriptomic, and clinical datasets analyzed in this study were obtained from publicly accessible repositories. The log2-normalized FPKM expression matrix of gastric cancer samples and the corresponding clinical and phenotype annotations from The Cancer Genome Atlas (TCGA-STAD) were downloaded from the UCSC Xena browser (https://xenabrowser.net/datapages/). Microarray expression datasets, including GSE13861, GSE26899, GSE26901, and GSE28541, were retrieved from the GEO database (https://www.ncbi.nlm.nih.gov/geo/). Publicly available single-cell RNA-sequencing datasets were obtained from GEO, including GSE134520, GSE150290, GSE163558, GSE167297, and GSE183904. An additional gastric cancer single-cell dataset (HRA000051) was downloaded from the Genome Sequence Archive (GSA; https://ngdc.cncb.ac.cn/gsa-human/browse/HRA000051).
