# Supplementary material for: Integrative single-cell atlas unveils heterogeneity and prognostic value of cancer-associated fibroblasts in gastric cancer
Source: Front Oncol. 2026 Jan 9;15:1559489. doi: 10.3389/fonc.2025.1559489 (PMC12827549; doi:10.3389/fonc.2025.1559489)
Supplement: Supplementary file 1 [file Image1.pdf]

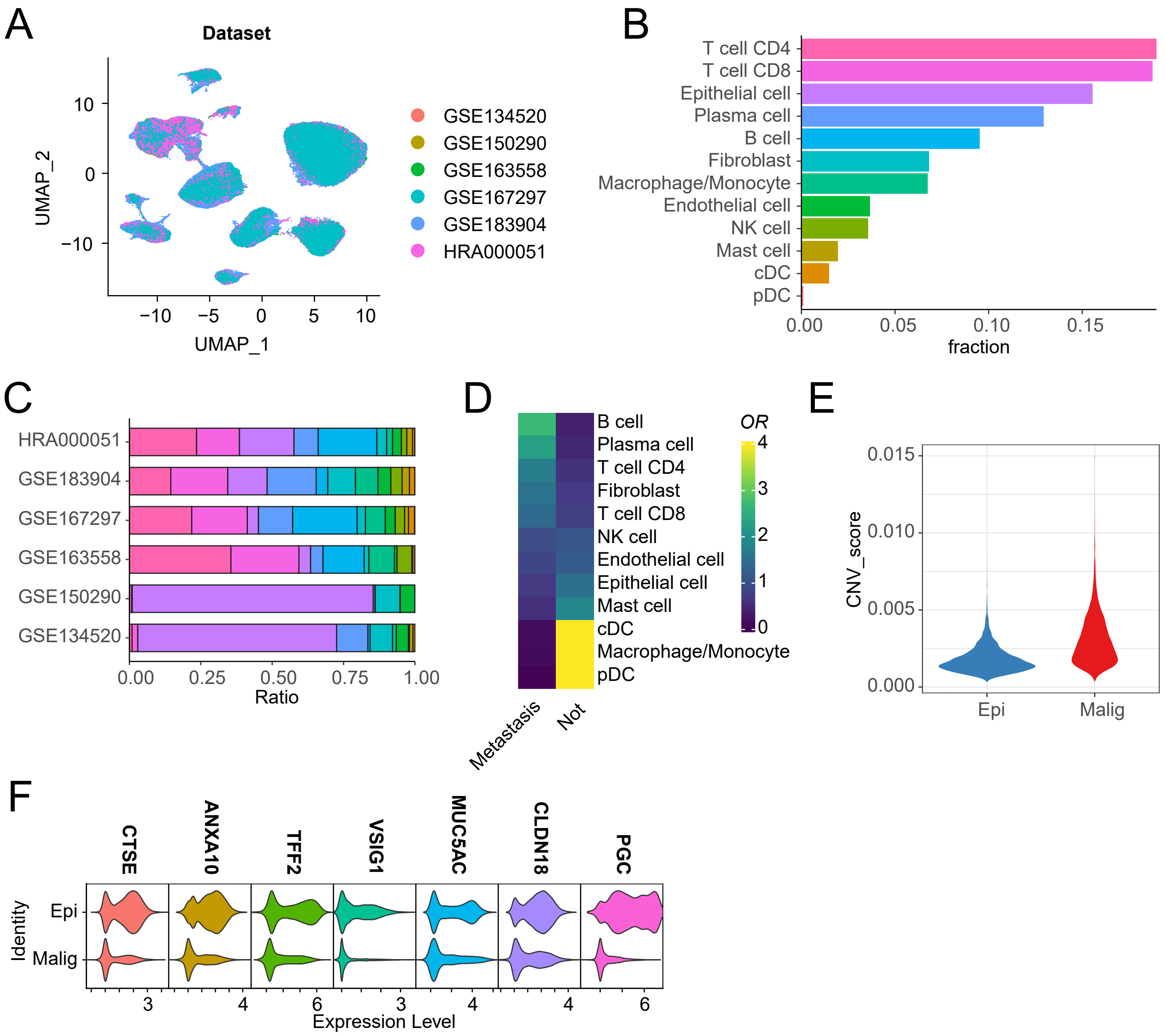

Figure S1. Composition of the gastric cancer single-cell atlas. (A) UMAP of core gastric cancer atlas from different datasets. (B) Cell type fractions in the core atlas. (C) Fractions of cell types and sample origins per study. (D) Cell-type composition between primary tumor and metastasis. (E) CNV score difference between normal epithelial cells and malignant cells. (F) Expression levels of gastric marker genes between normal epithelial cells and malignant cells.

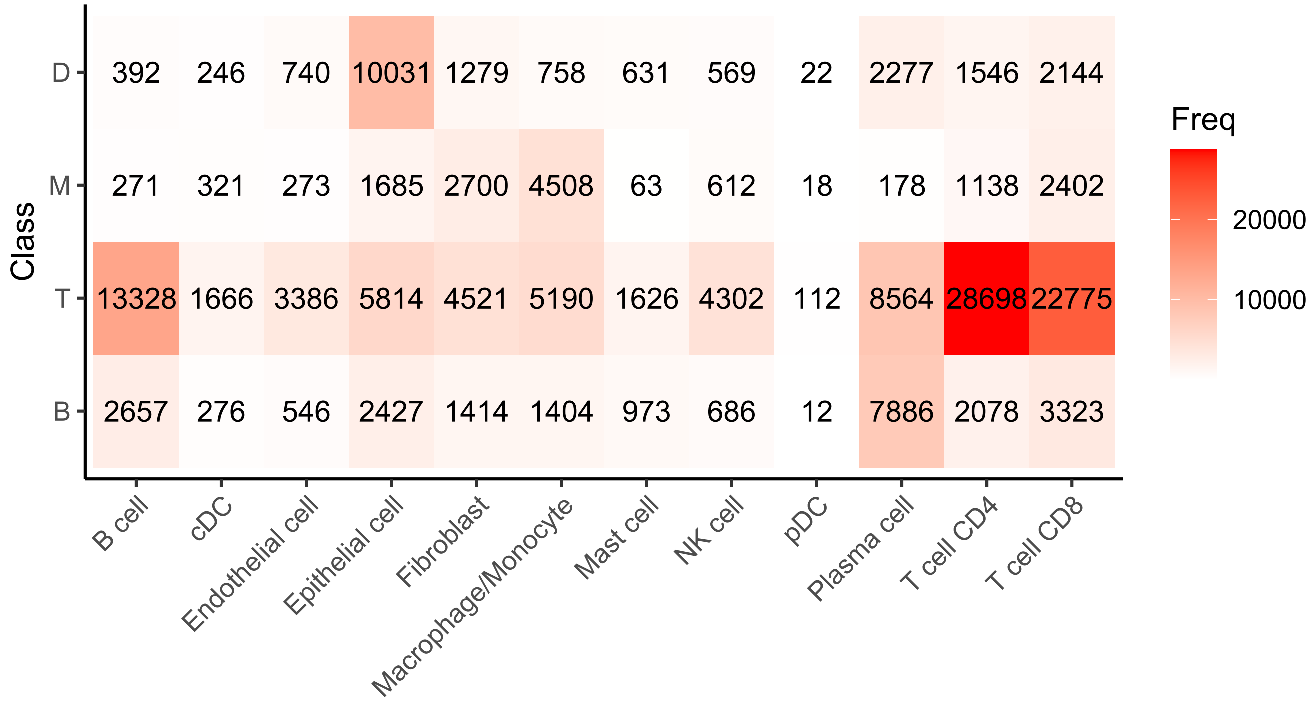

Figure S2. Tumor microenvironment subclusters by the contributing datasets. Left column shows the tumor microenvironment subclusters, the heatmap depicts the number of cells per subclusters.

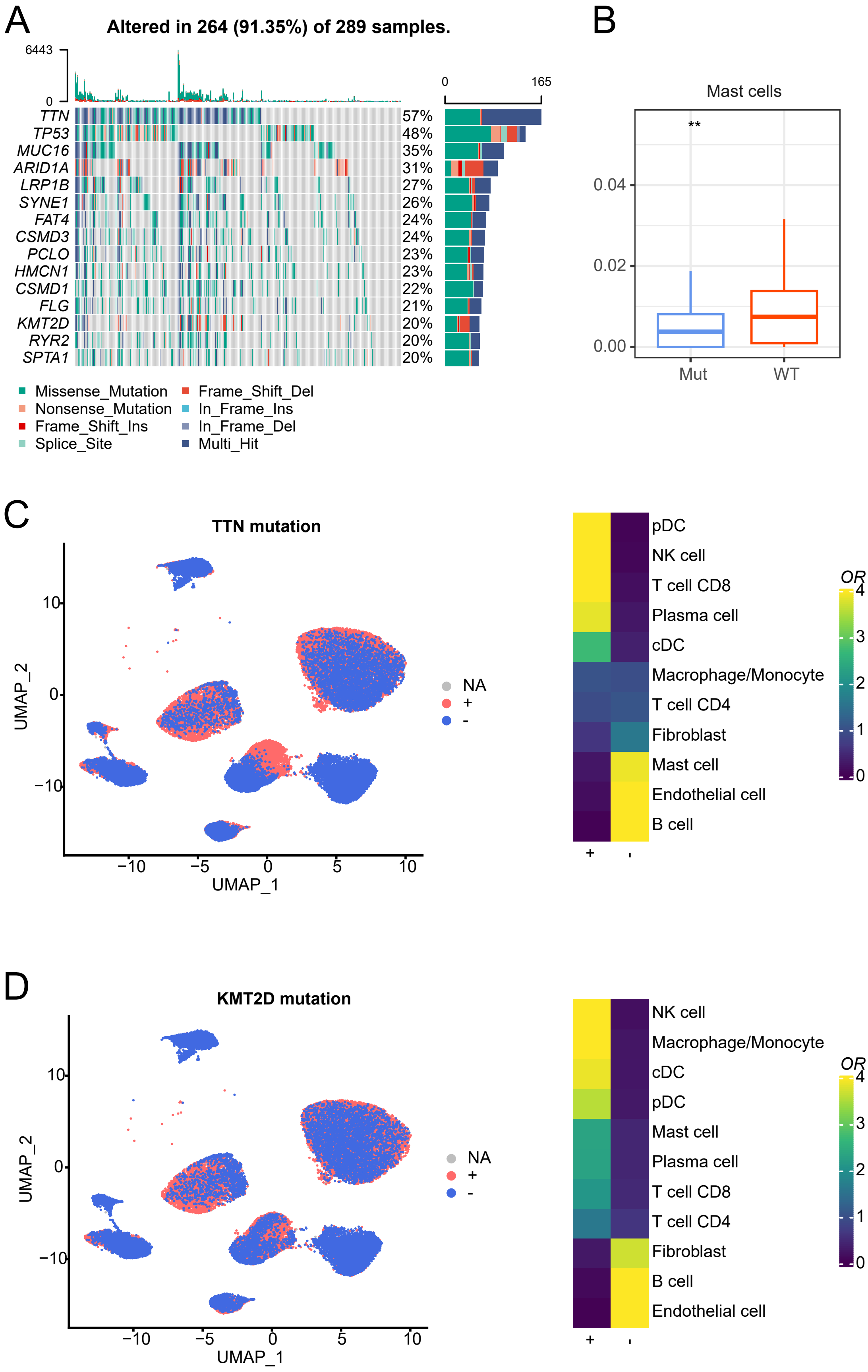

Figure S3. Association of cellular composition and distinct genotypes and survival in the TCGA data. (A) Mutation landscape of TCGA gastric cancer samples. (B) Cell type fractions difference between ARID1A mutation and wild-type samples. (C) Association of cellular composition with TTN mutation in patients with STAD. (D) Association of cellular composition with KMT2D mutation in patients with STAD.

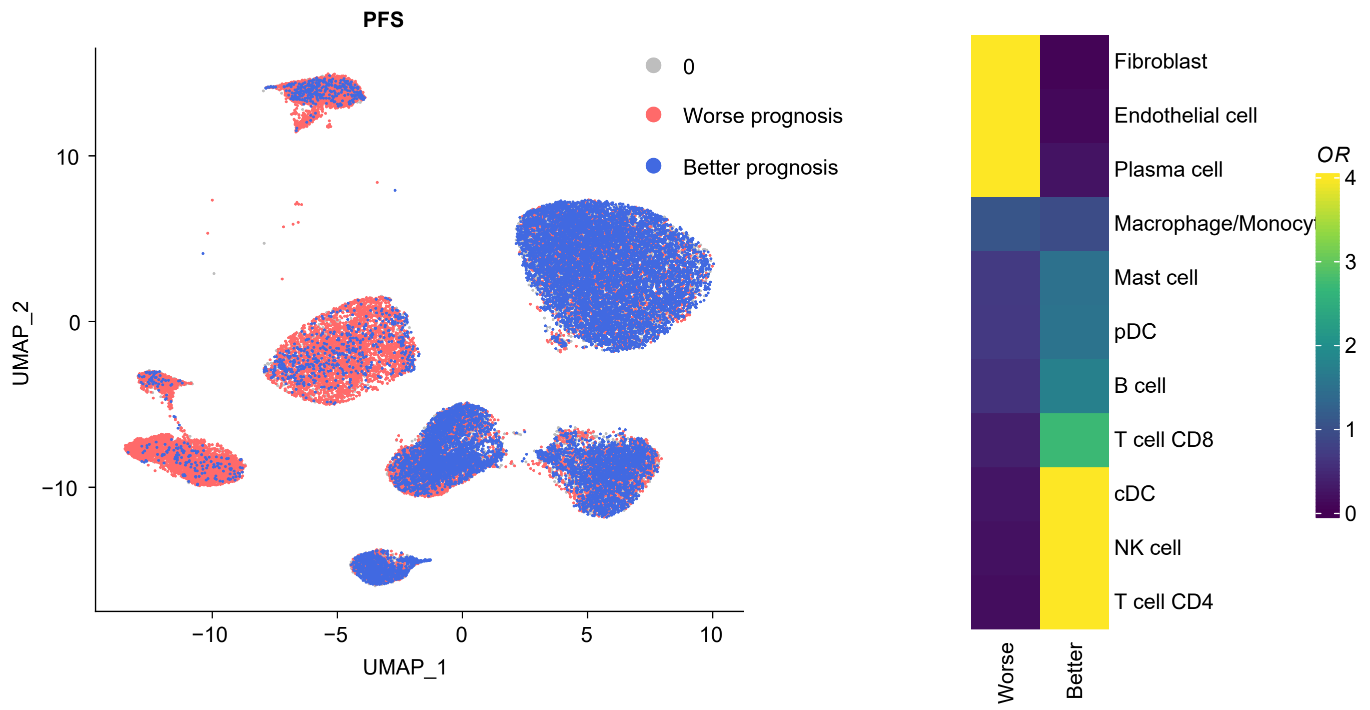

Figure S4. Association of cellular composition with progression free survival.

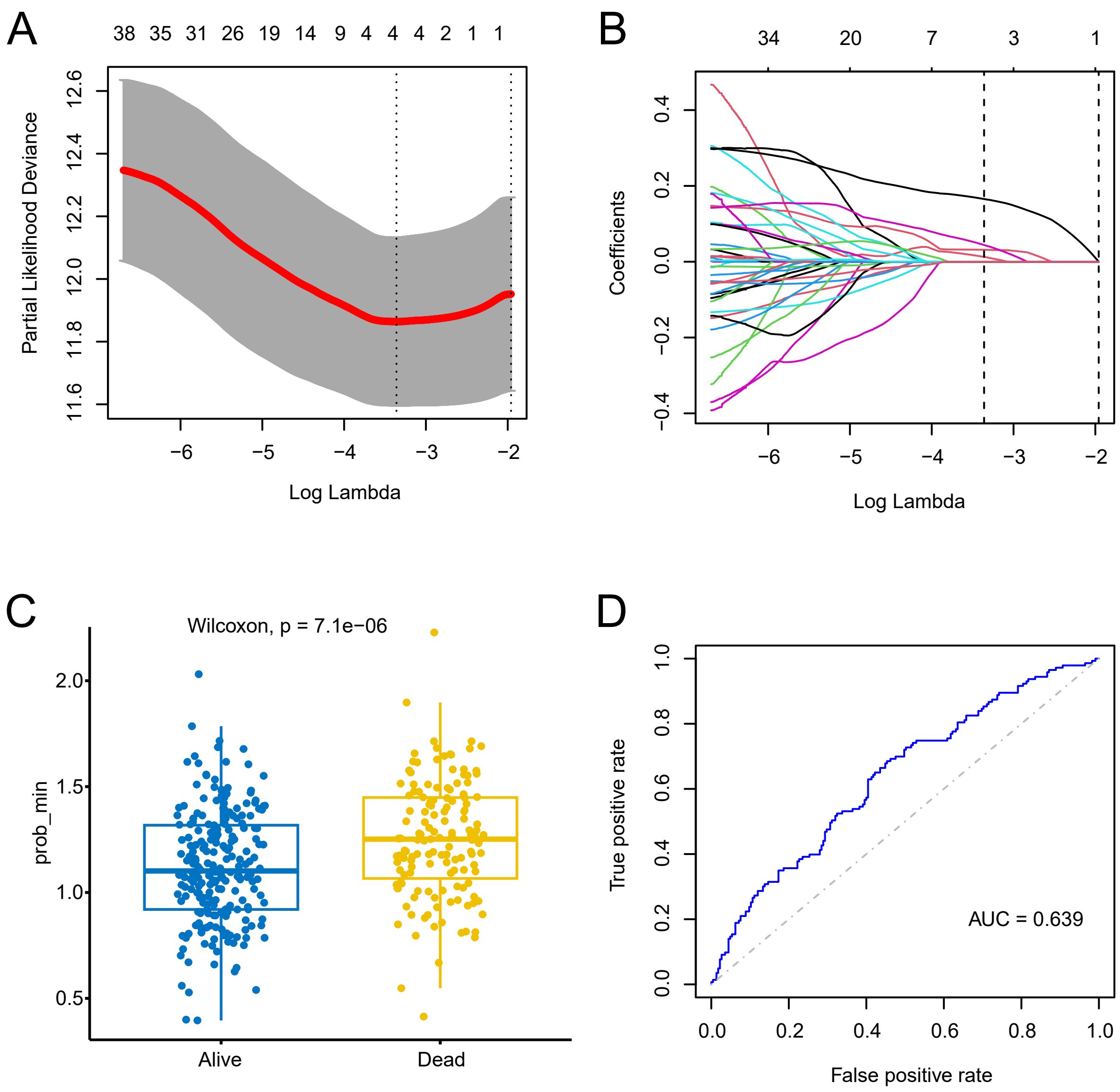

Figure S5. Marker gene identification and model construction. (A) The partial likelihood deviance is calculated by multivariate Cox regression. (B) The regression coefficients were calculated by multivariate Cox regression. (C) Boxplot shows the risk score difference between samples with different OS statuses. (D) ROC curve of risk scores and OS status.

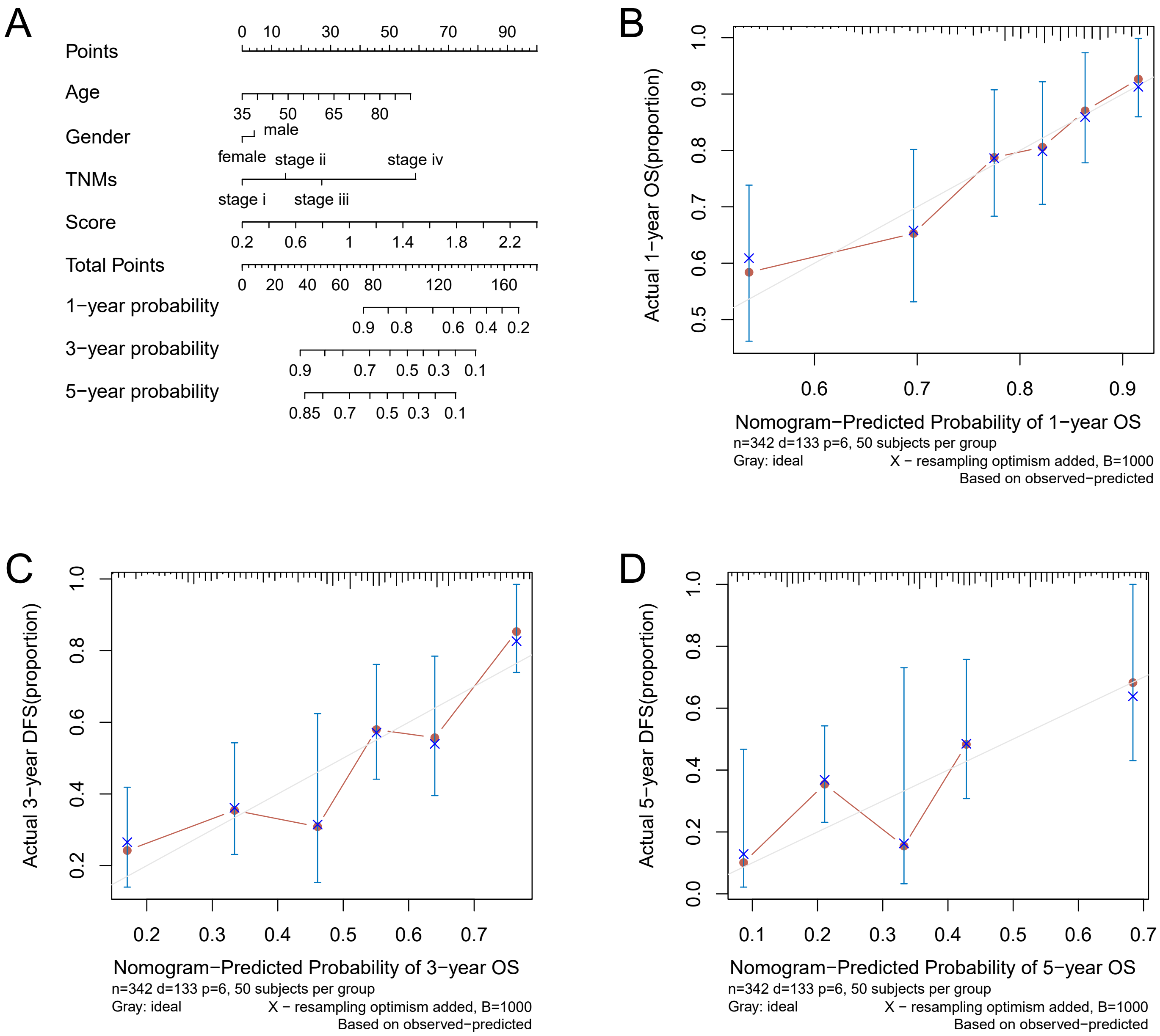

Figure S6. Nomogram analysis. (A) Nomogram composed of age, gender, TNM stage and risk score for the prediction of 1-, 3-, and 5-years OS probability. Calibration plot for the evaluation of the nomogram in predicting 1-year (B), 3-years (C), and 5-years (D) OS probability.
